# Strengthening the perception-assessment tools for dengue prevention: a cross-sectional survey in a temperate region (Madeira, Portugal)

**DOI:** 10.1186/1471-2458-14-39

**Published:** 2014-01-15

**Authors:** Teresa Nazareth, Rosa Teodósio, Graça Porto, Luzia Gonçalves, Gonçalo Seixas, Ana Clara Silva, Carla Alexandra Sousa

**Affiliations:** 1 GABBA Doctoral Program, ICBAS, Abel Salazar Institute for the Biomedical Sciences, University of Porto, Porto, Portugal; 2 Unidade Clínica Tropical, Instituto de Higiene e Medicina Tropical, Universidade Nova de Lisboa, Lisboa, Portugal; 3 Unidade de Parasitologia Médica, Instituto de Higiene e Medicina Tropical, Universidade Nova de Lisboa, Lisboa, Portugal; 4 Centro de Malária e Doenças Tropicais, Instituto de Higiene e Medicina Tropical, Universidade Nova de Lisboa, Lisboa, Portugal; 5 IBMC, Institute for Molecular and Cellular Biology, Porto, Portugal; 6 Unidade de Saúde Pública e Internacional e Bioestatística, Instituto de Higiene e Medicina Tropical, Universidade Nova de Lisboa, Lisboa, Portugal; 7 Centro de Estatística e Aplicações da Universidade de Lisboa (CEAUL), Universidade de Lisboa, Lisboa, Portugal; 8 Departamento de Saúde, Planeamento e Administração Geral, Instituto de Administração da Saúde e Assuntos Sociais, IP-RAM, Funchal, Portugal; 9 Unidade de Parasitologia e Microbiologia Médica, Instituto de Higiene e Medicina Tropical, Universidade Nova de Lisboa, Lisboa, Portugal

**Keywords:** Dengue prevention, *Aedes aegypti* control, Community involvement, Health education, Community-based participatory research, Community perception, Awareness and perception assessment, Domestic breeding sites, Behavioural change models, Knowledge-attitudes-and-practices surveys

## Abstract

**Background:**

Community participation is mandatory in the prevention of Dengue outbreaks. Taking public views into account is crucial to guide more effective planning and quicker community participation in preventing campaigns. This study aims to assess community perceptions of Madeira population in order to explore their involvement in the *A. aegypti*’s control and reinforce health-educational planning. Due to the lack of accurate methodologies for measuring perception, a new tool to assess the community’s perceptions was built.

**Methods:**

A cross-sectional survey was performed in the Island’s *aegypti-*infested area, exploring residents’ perceptions regarding most critical community behaviour: *aegypti*-source reduction and their domestic *aegypti*-breeding sites. A novel tool defining five essential topics which underlie the source reduction’s awareness and accession was built, herein called Essential-Perception (EP) analysis.

**Results:**

Of 1276 individuals, 1182 completed the questionnaire (92 · 6%). EP-Score analysis revealed that community’s perceptions were scarce, inconsistent and possibly incorrect. Most of the population (99 · 6%) did not completely understood the five essential topics explored. An average of 54 · 2% of residents only partially understood each essential topic, revealing inconsistencies in their understanding. Each resident apparently believed in an average of four false assumptions/myths. Significant association (p<0.001) was found between both the EP-Score level and the domestic presence of breeding sites, supporting the validity of this EP-analysis. *Aedes aegypti’s* breeding sites, consisting of décor/leisure containers, presented an atypical pattern of infestation comparing with dengue prone regions.

**Conclusions:**

The studied population was not prepared for being fully engaged in dengue prevention. Evidences suggest that EP-methodology was efficient and accurate in assessing the community perception and its compliance to practices. Moreover, it suggested a list of myths that could persist in the community. This is the first study reporting an *aegypti*-entomological pattern and community’s perception in a developed dengue-prone region. Tailored messages considering findings of this study are recommended to be used in future campaigns in order to more effectively impact the community perception and behaviour.

## Background

*Aedes aegypti* is one of the most competent vectors of dengue, yellow fever and chikungunya viruses. Recent estimations suggest a global impact of 390 million dengue infections annually worldwide [1]. Since there are no vaccines or specific treatments for this arboviral infection, the reduction of vector density is one of the most straightforward strategies for its prevention. Furthermore, recent studies unravel the high cost-effectiveness of an active and continuous vector control as opposed to an answer to dengue outbreaks [2]. According to the World Health Organization (WHO), *A. aegypti’s* control is mainly achieved by source reduction of the vector through the elimination of the mosquito breeding sites [3]. Due to *A. aegypti’s* domestic ecological feature, their larvae preferably proliferate in small and artificial water-containers, placed inside or near human houses [4]. Therefore, community contribution is, undoubtedly crucial in dengue prevention and control [5,6]. Educational campaigns that inform and mobilize the local communities are often implemented in the infested areas. In most preventive campaigns, the community is asked to do *aegypti*-source reduction: to eliminate (cover, empty and/or remove) the most common domestic breeding sites. Abundant literature may be found reporting community-oriented educational interventions and assessments of community knowledge/attitudes/practices/perceptions/beliefs regarding dengue prevention, most of which are performed in tropical regions [7-14]. Even though the relevance of the latter issues is more and more recalled by important entities [15,16], most of the studies emphasize the need of new research approaches to explain and increase their commonly low efficacy [11-14,17,18]. Consequently, studies that suggest and/or test strategies that more effectively promote community behaviours and more accurately assess community perception, are of great need [19]. The ‘community perception’ term used here means “the collective views of a group of people (…) [perception] involves understanding/misunderstanding and discernment, and it includes a choice and action (…) [perception is also] the product of social interaction”, as stated by WHO [19].

In the past years, several viruses and vectors have significantly increased their geographic distribution as a result of globalization [20,21]. In 2005, *A. aegypti* specimens were recorded for the first time in Madeira, a temperate European island in the Atlantic [22]. Rapidly, the local health authorities promoted educational activities based on television/radio communications, informative flyers/posters distribution and ‘door-to-door’ interventions to achieve community compliance in the domestic control of *A. aegypti*[23]. In fact, despite these efforts, the mosquito population has thrived. Additionally, entomological studies reported high levels of resistance to DDT and pyrethroids in the local *A. aegypti* population [24].

In October 2012, less than one year after the beginning of this study, an outbreak of dengue was declared in the Island [25]. Currently, Madeira is at risk of becoming a dengue endemic territory. Also, being a highly touristic destination, it constitutes an open door for *A. aegypti* and/or dengue virus introduction into other temperate regions [26]. Moreover, non-tropical regions such as Europe and North America host *Aedes albopictus* another very competent arboviral vector [27-29]. A unique virus introduction into these temperate regions could trigger a disease epidemic [30]. Community-mobilization strategies that effectively reduce *A*. a*egypti’s* densities in Madeira Island are thus, mandatory.

This study aims to estimate the community’s perceptions of Madeira residents regarding source reduction, and identify the most frequent *aegypti*-breeding sites present in the domestic environment of this non-tropical region. An extensive and in-depth analysis is suggested as a novel tool for community perception assessment and educational planning.

## Methods

### Studied population

The study area was chosen according to the *A. aegypti’*s distribution area, assessed by an island-wide entomological survey (Additional file 1). Based on mosquito abundance levels, a more restrictive zone called ‘AEGYPTI’, was selected. This area includes part of three municipalities: Santa Luzia and São Pedro (both in Funchal county), and Câmara de Lobos (in a Funchal neighbouring county). A representative sample of residents aged 18 years old or over was selected from the electoral system database, using stratified sampling by the municipality. A universe of 13 433 adult subjects lived in the area of study (almost 7% of the Island’s adult total population) [31]. A sample size of 1083 subjects, was required to fulfil the objectives of this study (90% confidence level and 2 · 5% precision). A prevalence of 50%, regarding good knowledge, was assumed. This sample size was inflated in 20% to account for non-respondents and incomplete interviews. Individuals who were not found or who refused to participate were replaced.

### Questionnaire and entomological inventory

A cross-sectional survey was performed through face-to-face interviews. In each interview, both a questionnaire to assess the residents’ perceptions and a domestic breeding site inventory of each household, were fulfilled. The surveys were performed by trained personnel (Health technicians of the local authority-IASAUDE) during October and November 2011. A total of three attempts were undertaken to contact the selected individuals: (i)-on weekdays between 9 am and 5 pm; (ii)-on weekdays between 5 pm and 8 pm; and (iii)-on Saturdays between 10 am and 7 pm. Participants gave oral informed consent prior to data collection. Previous to its application, the questionnaire was pre-tested in an *aegypti*-infested but non-selected area. The questionnaire comprised 13 questions, addressing five main topics (see criteria in Perceptions Evaluation paragraph): ‘Medical Importance’ (two questions), ‘Local Risk’ (two questions), ‘Domestic Attribute’ (three questions), ‘Mosquito Breeding’ (three questions) and ‘Control Measures’ (three questions). The questionnaire also covered socio-demographic characteristics. The breeding site inventory listed 21 types of putative domestic breeding sites present in each household. The study was approved by *Instituto de Higiene e Medicina Tropical Ethics Committee* (reference: 09-2013-TD).

### EP-analysis (Perception evaluation)

The most common answer frequency estimation was calculated (data not shown).

However, in order to accomplish accurate and in-depth perception estimation, several analysis were performed.

A list of five essential topics regarding source reduction was defined. Topics correspond to variables known to determine behaviour changes, such as, self-efficacy, behavioural expectancies, perceived susceptibility, etc. as mentioned in several models of behavioural change described in the literature [18,32]. According to behavioural change experts, the list of variables/topics were chosen and adapted to dengue context and to the particular Madeira scenario [18,32]. The five selected variables (here called ‘topics') are individually labelled as: (*A. aegypti*’s) Medical Importance, (its) Local Context, Domestic Attribute (of its vector-control), Mosquito Breeding (process) and finally, (vector)-Control Measures. We established the awareness and the understanding of these five topics as necessary and obligatory for the acceptance of (and presumed consequent adherence to) source reduction practice.

Two concepts were selected to evaluate each of the latter five topics (these are here called ‘Essential concepts’). By evaluating the acknowledgement of both Essential concepts, a double-evaluation of the understanding of each of the five topics was done. This allowed for the detection of discrepancies in the way these five topics are understood. Collectively the ten concepts sum-up the awareness of the source reduction. This way, this methodology allows the estimation of the community’s perceptions through four distinct approaches: (i)-score of Essential-Perception, (ii) concept's assimilation, (iii) topic understanding and (iv)-discrepancy detection/myth estimation, all described below.

#### Concepts assimilation and score of essential-perceptions (EP-score)

According to the residents’ answers, the acknowledgement of the ten essential concepts was calculated. Each concept corresponds to one or two questions. We obtained the EP-score for each resident assimilated (from 0 to 10), by attributing one point to each perceived essential concept. Thus, EP-score level corresponds to the number of (essential) concepts, out of the ten established that each resident has assimilated. Following EP-analysis’ criteria, only those who achieved an EP-score equal to 10 showed minimal and adequate perceptions to trigger individual compliance in source reduction (see an example in Additional file 2). Respondents who have not answered all the 13 questions were excluded from score calculation.

#### Topic understanding

The understanding of the five covered topics was evaluated according to the knowledge shown in topic-related essential concepts (Graphic 1 and 2). Only residents who had acknowledged both topic-related concepts had completely understood the topic. The acknowledgement of only one out of the two topic-related concepts revealed a partial understanding. Residents who did not perceive any of the two topic-related concepts did not understand the topic.

#### Discrepancy detection/myths estimation

Partial or absent understanding of one of the five topics could generate false perceptions concerning it (Additional file 3). By analyzing the acknowledgement of both Essential concepts for each topic and the discrepancies in its understanding, a list of myths (false information that is perceived as true by a part of the population) was estimated and also its supposed frequency in the population (Additional file 4).

### Statistical analysis

All collected information was introduced and records were double-checked. Statistical analysis was performed using Excel (Microsoft Office, Windows Vista) and Statistical Package for Social Sciences 19.0 (SPSS, Inc., Chicago, IL, USA). Answers obtained from the questionnaire were re-coded to obtain other categorical variables linked to the above mentioned ten concepts. Determinants of the EP-Score level and predictors of the domestic presence of breeding-sites were also explored. EP-Score percentiles for each socio-demographic group were calculated following Weighted Average method. Comparisons of score medians between socio-demographic groups were made using non parametric tests: Mann–Whitney and Kruskal-Wallis. Associations/differences with the domestic presence of breeding sites were performed using three different approaches: (i)-individual essential concepts: assessed by a chi-square test for categorical variables; (ii)-EP-Score: assessed by Weighted Averaged method and Mann–Whitney test; (iii)-Incomplete Scores (four combinations of scores covering four out of the five main topics) also assessed by Weighted Averaged method and Mann–Whitney test. In this latter point (iii), by filtering the residents that showed zero points regarding each of the five topics separately, four combinations of incomplete EP-Scores (from 0 to 8 points) were generated. Additionally, logistic regression models were also performed to explore socio-demographic factors that contribute to achieve, or not, an EP-Score equal to or higher than seven. The cut-off would preferably be an EP-Score equal to 10 (instead of 7). However, due to the inexistence of a minimum number of individuals that have reached the maximum (EP = 10), the cut-off was adjusted until 7 in order to include a enough number of individuals needed to perform the logistic regression.

## Results

A total of 1276 AEGYPTI-residents participated in the study. Out of these, only 92 · 6% (1182 individuals) answered the 13 questions and were scored according to the perceptions demonstrated. All individuals’ residences were inventoried to putative breeding sites. Table 1 shows the socio-demographic characteristics of the studied population.

**Table 1:** Socio-demographic characterization of the inquired / scored population and EP-Score results per socio-demographic groups

	**Inquired population (n = 1276)**	**Scored population (n = 1182)**		
	**n**	**n (%)**	**EP-score median (P**_**25**_**-P**_**75**_**)**^**+**^	***p*-value**
**Gender (n = 1267)**				<0 · 001‘
Male	506	480 (40 · 6)	5 · 0 (4 · 0 - 7 · 0)	
Female	761	701 (59 · 4)	5 · 0 (3 · 0 - 6 · 0)	
**Education level (years) (n = 1251)**				<0 · 001‘’
Never studied (0)	75	69 (5 · 9)	3 · 0 (2 · 0 – 4 · 0)	
Fourth grade (4)	484	446 (38 · 2)	4 · 0 (3 · 0 – 5 · 0)	
Ninth grade (9)	281	262 (22 · 5)	5 · 0 (4 · 0 – 6 · 0)	
High school (12)	220	207 (17 · 7)	6 · 0 (4 · 0 – 7 · 0)	
Upper education (+12)	191	183 (15 · 7)	7 · 0 (6 · 0 – 8 · 0)	
**Age groups (years) (n = 1256)**				<0 · 001‘’
25 or younger	170	154 (13 · 2)	4 · 0 (3 · 0 – 6 · 0)	
26-35	172	161 (13 · 8)	5 · 0 (3 · 0 – 7 · 0)	
36-45	197	191 (16 · 3)	5 · 0 (4 · 0 – 7 · 0)	
46-55	221	207 (17 · 7)	5 · 0 (4 · 0 – 7 · 0)	
56-65	182	174 (14 · 9)	5 · 0 (3 · 0 – 6 · 0)	
66-75	185	167 (14 · 3)	5 · 0 (3 · 0 – 6 · 0)	
76 or older	129	116 (9 · 9)	4 · 0 (3 · 0 – 6 · 0)	
**Municipality (n = 1275)**				<0 · 001‘’
Santa Luzia	417	388 (32 · 9)	6 · 0 (4 · 0 – 7 · 0)	
São Pedro	314	304 (25 · 7)	5 · 0 (4 · 0 –7 · 0)	
Câmara de Lobos	544	489 (41 · 4)	4 · 0 (3 · 0 – 5 · 0)	
Travelled to EC* (n = 1245)				<0 · 001‘
Yes	311	287 (24 · 7)	5 · 0 (4 · 0 – 7 · 0)	
No	934	876 (75 · 3)	5 · 0 (3 · 0 – 6 · 0)	
**‘Bitten by mosquitoes’ (n = 1271)**				
Yes	944	887 (75 · 2)	5 · 0 (4 · 0 – 7 · 0)	<0 · 001‘
No	327	293 (24 · 8)	4 · 0 (3 · 0 – 6 · 0)	

Some descriptive statistics (percentages, median, and percentiles) illustrate the socio-demographic feature and EP-score results. Comparisons of EP-score’s medians between socio-demographic groups are also presented *(p*-values). Not all the respondents answered to all the socio-demographic questions, thus correspondent n values are described.

^+^Weighted Average method; ‘Mann–Whitney test; ‘’Kruskal-Wallis test.

### EP-analysis

#### EP-score and concepts assimilation

**Figure 1:**
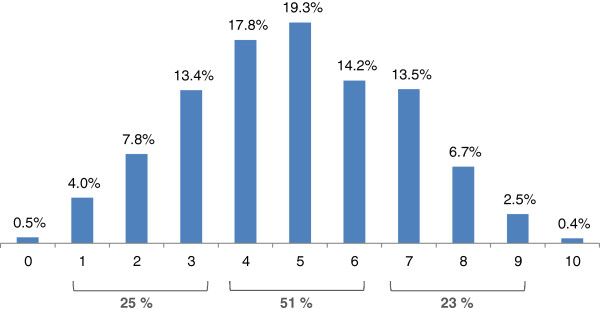
Proportion of respondents that achieved each EP-Score’s levels (in percentage, n Total = 1182).

Respondents’ EP-score distribution is represented in Figure 1. Only 0 · 4% out of the scored respondents (5 individuals) achieved an EP-score = 10. The total population recognized an average of five essential concepts, half of those evaluated.

**Figure 2:**
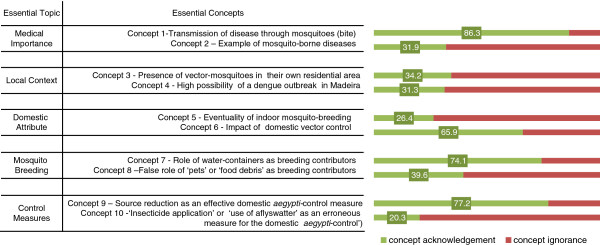
Proportion of respondents that acknowledged each Essential Concept.

Population acknowledged the ten essential concepts differently (Figure 2). The concepts ’Medical Importance 1’ and ‘Control Measures 1’ were the most well-acknowledged; 86 · 3% of the interviewed admitted that mosquitoes can transmit diseases (MI1-concept) and 77 · 2% referred to the reduction of breeding sites as being a “(fairly/very/extremely) effective measure” in controlling mosquitoes (CM1-concept). On the contrary, concepts ‘Control Measures 2’ and ‘Domestic Attribute 1’ were the least recognized; only 26 · 4% acknowledged that “mosquitoes can breed inside houses” (DA1-concept) whereas only 20 · 3% of the studied population correctly admitted to CM2-concept which did not identifying the use of a flyswatter or indoor insecticide spraying, as effective for *aegypti*-control.

#### Topic understanding

**Figure 3:**
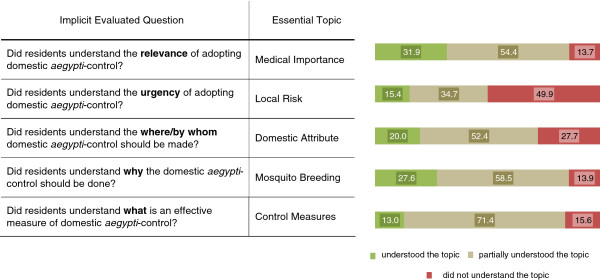
Proportion of respondents that ‘understood’, ‘partially understood’ and ‘did not understand’ each Essential Topic.

Regarding the topics, shown in Figure 3, ‘Medical importance’ was the one that more people have completely understood (31 · 9% of the studied population), while both the concepts related to ‘Control Measures’ were only recognized by 13 · 0% of the respondents. By analysing each topic separately, Graphic 3 reveals that the majority of the respondents presented partial understanding of four out of the five topics. Differently, for ‘Local Risk’ the highest proportion of the respondents disregarded both topic-related concepts.

#### False perceptions/myths estimation

Based on the analysis of AEGYPTI-residents topics understanding a list of 13 alleged myths was elaborated and its supposed frequency in the population calculated (Table 2). The most disseminated myth was: “the insecticide usage as an effective measure to control *aegypti*-mosquitoes” found in 79 · 7% of the scored population. Each resident believed, on average, in 4 out of the 13 myths. Most of them (99 · 5%) believed at least in one myth (Table 2).

**Table 2:** List of the thirteen alleged myths and proportion of residents that believed in each of them

**Essential topic**	**Alleged myth**		**n (%)**
**Medical importance**	Myth 1	“Mosquitoes only cause mild clinical consequences such as allergies, fever, etc”.	643 (54 · 4)
	Myth 2	“Mosquitoes do not transmit diseases”.	162 (13 · 7)
**Local risk**	Myth 3 and Myth 4	“Dengue is not a mosquito-borne disease” and/or “Dengue only occur in tropical/non-developed countries”.	222 (18 · 8)
	Myth 5 and Myth 6	“Since I do not feel the byte, I am not at risk of being bitten/infected” and/or “Mosquitoes are allocated in a specific area and are not able to spread through the island”.	188 (15 · 9)
	Myth 7	“Madeira’s residents are not at risk”.	590 (49 · 9)
**Domestic attribute**	Myth 8	“Local health authorities are the key intervenient in the control of mosquitoes”.	76 (6 · 4)
	Myth 9	“Insecticides or other protective measures can control mosquitoes”.	543 (45 · 9)
	Myth 10	“I am (Community is) not an intervenient in the *aegypti*-control”.	590 (49 · 9)
**Mosquito breeding**	Myth 11 and Myth 12	“Clean houses or houses without pets/animals do not have mosquitoes” and/or “Clean people have nothing to do concerning the control of mosquitoes”.	714 (60 · 4)
**Control measures**	Myth 13	“By the usage of insecticides and/or flyswatter, I am already contributing to the *aegypti*-control”.	942 (79 · 7)

Based on the analysis of the discrepant knowledge showed concerning topic-related concepts, false assumptions/myths were inferred to be present in the scored population (see Myths’ estimation and Myth’s appearance on Additional files).

Average of believed myths per scored resident: four out of the thirteen myths.

Proportion of scored residents that believed in at least one alleged myth: 99 · 5%.

### Entomological description, its determinants and correlations with perceptions

Out of all the 1276 interviewed individuals 79 · 6% lived in houses with at least one putative breeding site. The most frequent breeding sites were: flower-pot dishes, present in 52 · 7% of the respondent’s houses; out-door sinks (35 · 7%); water-accumulation on decks (23 · 3%); flower vases (21 · 7%) and pet water-dishes (18 · 8%) (Additional file 5).

Statistical tests were performed in order to explore whether or not the presence of breeding sites were determined by the EP-Score level. According to Table 3, no significant differences were found between those that admitted/not admitted to concepts ‘Mosquito Breeding1’ and ‘Control Measures 1’. However, residents who had breeding sites in their households had significantly lower EP-scores compared to those living in houses without breeding sites (Table 3). Comparing the five ‘Incomplete Scores’ within both of the residents’ houses with/without domestic breeding sites, none of the five combinations varied significantly (see Table 4). Municipality also presented significant association with the presence of domestic breeding sites, being ‘Santa Luzia’ the one with higher frequency of households without breeding sites (Additional file 6).

**Table 3:** Associations between the domestic presence of putative breeding sites (any type) and: (a) acknowledgement of concept ‘Mosquito Breeding1’; (b) acknowledgement of concept ‘Control Measure 1’ and (c) cumulative essential-concepts’ acknowledgement: EP-score

		**Residents living in houses…**				
		**…WITH breeding-sites**		**….WITHOUT breeding-sites**		
		**n (%)**	**median (P**_**25**_**-P**_**75**_**)**^**+**^	**n (%)**	**median (P**_**25**_**-P**_**75**_**)**^**+**^	***p*-value**
**(a) “Role of water-containers as breeding contributors (Concept 7)”**	Acknowledged	699 (73 · 4)	-	177 (77 · 0)	-	0 · 272ˇ
	Did not acknowledge	253 (26 · 6)	-	53 (23 · 0)	-	
**(b) “Source reduction as an effective domestic *aegypti-*control measure (Concept 9)”**	Acknowledged	728 (76 · 5)	-	184 (80 · 0)	-	0 · 253ˇ
	Did not acknowledge	224 (23 · 5)	-	46 (20 · 0)	-	
**(c) EP-score**		952 (80 · 5)	5 · 0 (3 · 0 – 6 · 0)	230 (19 · 5)	5 · 0 (4 · 0 – 7 · 0)	0 · 001‘

‘Mann–Whitney test; ˇPearson test; ^+^Weighted Average method.

**Table 4:** Association of EP-Incomplete Scores and presence of domestic breeding sites

**Essential topic excluded**	**Residents living in houses**	**Residents living in houses**	***p - *value**‘
	**WITH breeding-sites**	**WITHOUT breeding-sites**	
	**n; median (P**_**25**_**-P**_**75**_**)**^**+**^	**n; median (P**_**25**_**-P**_**75**_**)**^**+**^	
Medical importance	137 ; 2 · 0 (2 · 0 – 4 · 0)	25 ; 3 · 0 (1 · 0 – 4 · 0)	0.615
Local risk	484 ; 4 · 0 (3 · 0 – 5 · 0)	106 ; 4 · 0 (3 · 0 – 5 · 0)	0.399
Domestic attribute	267 ; 3 · 0 (2 · 0 – 4 · 0)	60 ; 3 · 0 (2 · 0 – 4 · 0)	0.515
Mosquito breeding	138 ; 3 · 0 (2 · 0 – 4 · 0)	26 ; 3 · 0 (1 · 0 – 3 · 0)	0.367
Control measures	155 ; 3 · 0 (2 · 0 – 3 · 0)	29 ; 2 · 0 (1 · 0 – 3 · 0)	0.351

Incomplete EP-score covered only four out of the five Essential Topics.

‘Mann–Whitney test; ^+^Weighted Average method.

### Socio-demographic characteristics and perception determinants

All analysed socio-demographic characteristics presented significant differences in EP-scores medians (Table 1). Actually, all males, residents aged 26–35 years old, people that had 12 years or more of education, individuals that live in ‘Santa Luzia’, respondents that have travelled to EC and those that admitted to have been bitten by mosquitoes, have embraced more essential concepts than their correspondent socio-demographic groups. Following the logistic regression, four socio-demographic characteristics significantly determined a minimum of seven acknowledged essential concepts (EP-Score equal to or higher than seven). These were residents’ ‘gender’, ‘municipality’, the eventuality of being ‘bitten by mosquitoes’ and above all ‘educational level’ (Additional file 7).

## Discussion

Comparing to other studies, analysis of single concept frequency revealed an (apparent) very good community knowledge [12,13]. For example, almost 80% of the population recognized that “the source reduction is an effective measure for domestic *aegypti*-control” (Control Measure 1). However, perception evaluation based on EP-score showed that several essential concepts are still unknown by the majority of the population. Regarding topics understanding, only a few respondents completely understood each of the five topics. In all of them, a great discrepancy was found within the knowledge shown in concepts covering the same topic, predicting the presence of alleged myths/erroneous perceptions in most of the AEGYPTI-population. As suggested in Additional file 3, the dissemination of part of the information can promote the advent of myths. To notice, through an anthropological view these myths are considered the real perception of the community [33]. They are here called ‘erroneous perceptions or myths’ since they oppose and contradict what, to date, is considered to be the main community vector-control practice. Sequential educational activities should take into account those myths given that they could be much harder to amend than the lack of awareness itself.

Four socio-demographic determinants were described in the logistic regression results. Similarly to other studies, the education level was the most relevant determinant in the EP-Score level above 7, emphasizing the relevance of extensive health education programs to improve the health-literacy levels [34-37]. The ‘bitten by mosquitoes’ variable (stating the recognition of having been bitten by mosquitoes) also showed to be a determinant in the level of EP-Score. These suggests that measures that make the problem more ‘visible’ would be of a great impact in community awareness, especially for those who lack the allergic reaction to the bite. Determinants such as, ‘Gender’, and ‘Municipality’ should be considered in the selection of target groups/areas for further campaigns.

Concerning the entomological survey, only putative breeding sites were inventoried. Due to the un-expected absence of rainfall during the period of the study (carried-out during the beginning of the rainy season), most of the containers were dry (Additional file 8). Nevertheless, this was, to our knowledge, the sole entomological survey in a temperate region describing the most common *A. aegypti*’s domestic breeding sites. The most inventoried putative breeding sites were housing-components present in any patio, balcony or garden areas. An *aegypti*-infestation pattern was observed compatible with a clean, organized and well maintained urban environment (as schematized in Additional file 9). These results contrast with the common symbols of mosquito infestation in dengue endemic regions, often related to water supply and waste disposal (tires, water tanks, etc.) [38-40]. ‘Santa Luzia”s municipality showed a significantly higher percentage of houses without breeding sites compared to the other two municipalities. This could be explained by a higher conscience of the *A. aegypti*’s presence in ‘Santa Luzia’ since it was where this mosquito first appeared.

Associations found between EP-Score and presence of domestic breeding sites supported the established criteria (Tables 3 and 4). The important and most acknowledged concepts: DA2 and CM1, *per se* did not correlate with the absence of breeding sites. Yet, the EP-score level is significantly higher in respondents living in households without putative breeding sites (Table 1). These results seem to support that essential-concepts’ cumulative assimilation is needed for triggering the adoption of the aimed behaviour. Moreover, results from the Incomplete Scores revealed that none of the five topics were dispensable in the improvement of the source reduction compliance. Evidence was provided to use the EP-Score analysis as an accurate tool for perception estimation. Furthermore, comparing to the alternative simple analysis of frequencies (see Table 3), this tool provides deeper and more precise results to explore the community involvement. Actually, the major limitation of knowledge/perception assessments is the lack of its correlation with the adoption of proposed practices, frequently observed in similar studies (most commonly, knowledge-attitudes-and-practices surveys) [10,14,15,34-36,41]. Methodologies that estimate awareness based on a score were already used in other surveys [13,14]. However, these approaches rarely or never focus on a specific behaviour, and almost never test understanding discrepancies. Since the adoption of different dengue-related practices (preventing, protecting, diagnosing, treatment-seeking practices, etc.) implicates the understanding of distinct concepts, behaviour-oriented approaches are much more useful to prioritize health-messages and plan campaigns [41]. Analysis of discrepancies in the understanding has been suggested as a way to improve reliability in KAP surveys [17]. Similar studies are now needed to confirm whether this approach is indeed more accurate to assess perceptions and more effective to promote behaviours in the community.

## Conclusions

After seven years of coexistence with the *A. aegypti,* Madeira Island presents an atypical scenario of domestic infestation. Subsequent to several local educational activities, AEGYPTI-community perceptions regarding source reduction were not only insufficient, but also, inconsistent and possibly incorrect. Findings of this study provide crucial guidelines for future educational activities. By addressing the less acknowledged essential concepts and the alleged myths, and by emphasizing the most frequent breeding sites, health messages adapt their content and their focus to more likely help the community in fully engaging in the proposed behaviour. However, after the experience of a dengue outbreak (2012), local population has probably altered their perception, namely in what concerns the topic ‘Local Risk’. Moreover, since, no hemorrhagic clinical cases were detected in the latter outbreak, the real ‘Medical Importance’ of dengue could be still underestimated. These ideas should also be considered by those planning further educational activities on the island. As part of future actions the implementation of another questionnaire, similar to the one carried-out in this study, should be encouraged. In reality, with its recent dengue event, Madeira Island presents an exceptional opportunity to understand the effect of a disease-outbreak in a community’s awareness. Finally, findings of this study support the use of EP-Score methodology as a more efficient tool to evaluate the community-perception regarding a specific behaviour. When further tested, this type of tool will probably prove to be of great value for other health problems, far beyond dengue prevention.

## Abbreviations

WHO: World Health Organization; EP-score: Score of essential-perception; AEGYPTI: Madeira’s geographical area of major *A. aegypti*’s abundance levels at 2011 and selected for the present survey; KAP: Knowledge attitudes and practices.

## Competing interests

The authors declare that they have no competing interests.

## Authors’ contributions

TN and GS collectively worked in data collection and made literature search. Study-design was elaborated by TN, CAS, RT, GP and ACS. Statistical analysis was performed by TN and LG. Latter authors together with CAS, RT and GP worked on data interpretation and writing. All authors read and approved the final manuscript.

## Pre-publication history

The pre-publication history for this paper can be accessed here:

http://www.biomedcentral.com/1471-2458/14/39/prepub

## Supplementary Material

Additional file 1 ***A. aegypti*’s distribution area (2001).** Ovitrap distributions in the two inhabited island of Madeira’s archipelago: Madeira and Porto Santo (2011). Red Points correspond to positive ovitraps, Green Points correspond to negatives ones. Click here for file

Additional file 2 **Relevance of cumulative knowledge.** Exploring why a ‘higher’ level of knowledge doesn’t necessarily reflect a ‘better’ awareness. Click here for file

Additional file 3 **Myth’s appearance.** Explaining an example of how a myth can appear from a partial (non-cumulative) understanding. Click here for file

Additional file 4 False perceptions/myths estimation through the analysis of residents’ topic understanding. Click here for file

Additional file 5 **Domestic breeding sites.** Percentage (%) of inquired residents living in houses with each type of breeding site (n Total =1276). Click here for file

Additional file 6 **Domestic breeding sites predictors.** Associations/differences with socio-demographic data. Click here for file

Additional file 7 Multiple regression model predicting socio-demographic determinants to achieve at least seven perceived essential concepts (EP-score equal to or higher than seven). Click here for file

Additional file 8 Variation of the temperature, humidity and precipitation from September 2011 to July 2012 in Madeira Island. Click here for file

Additional file 9 **Representation of the *aegypti*-infestation pattern found in the domestic regions of AEGYPTI-area in Madeira Island.** Click here for file
