# Supplementary figures and images for: Strengthening the perception-assessment tools for dengue prevention: a cross-sectional survey in a temperate region (Madeira, Portugal)
Source: BMC Public Health. 2014 Jan 15;14:39. doi: 10.1186/1471-2458-14-39 (PMC3905660; doi:10.1186/1471-2458-14-39)

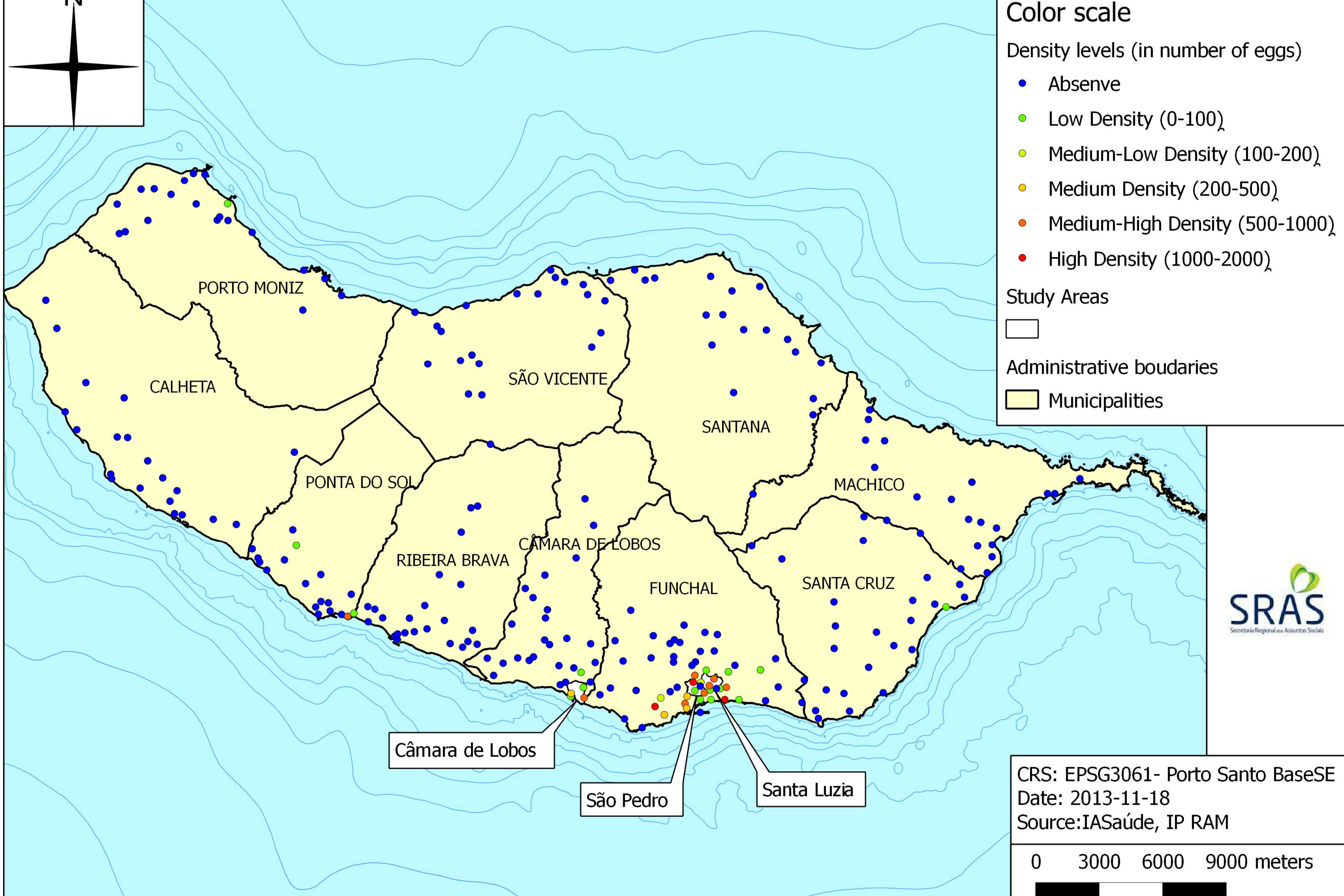

Supplement: Additional file 1 — A. aegypti’s distribution area (2001). Ovitrap distributions in the two inhabited island of Madeira’s archipelago: Madeira and Porto Santo (2011). Red Points correspond to positive ovitraps, Green Points correspond to negatives ones. [file 1471-2458-14-39-S1.pdf]

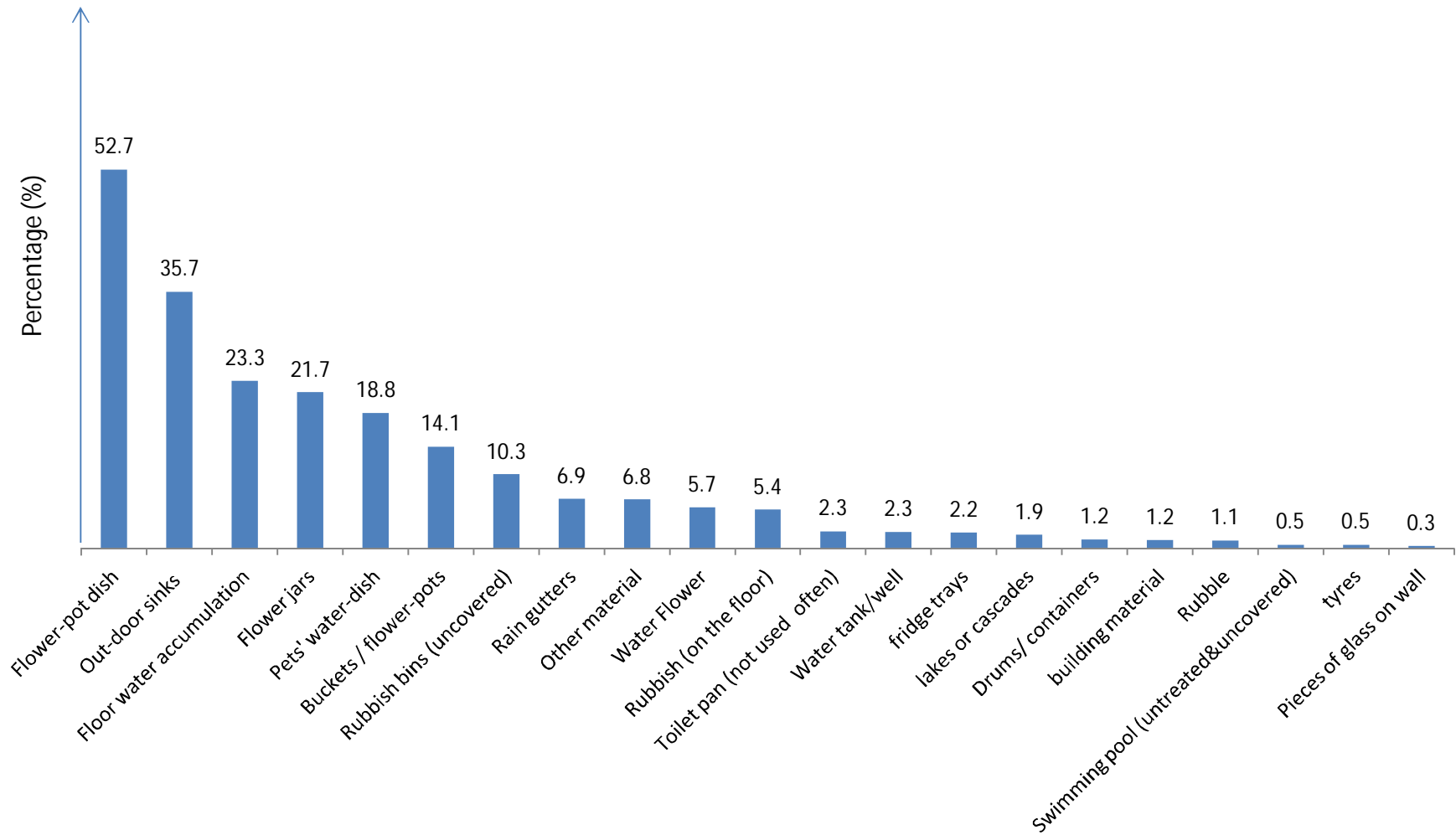

Supplement: Additional file 5 — Domestic breeding sites. Percentage (%) of inquired residents living in houses with each type of breeding site (n Total =1276). [file 1471-2458-14-39-S5.pdf]

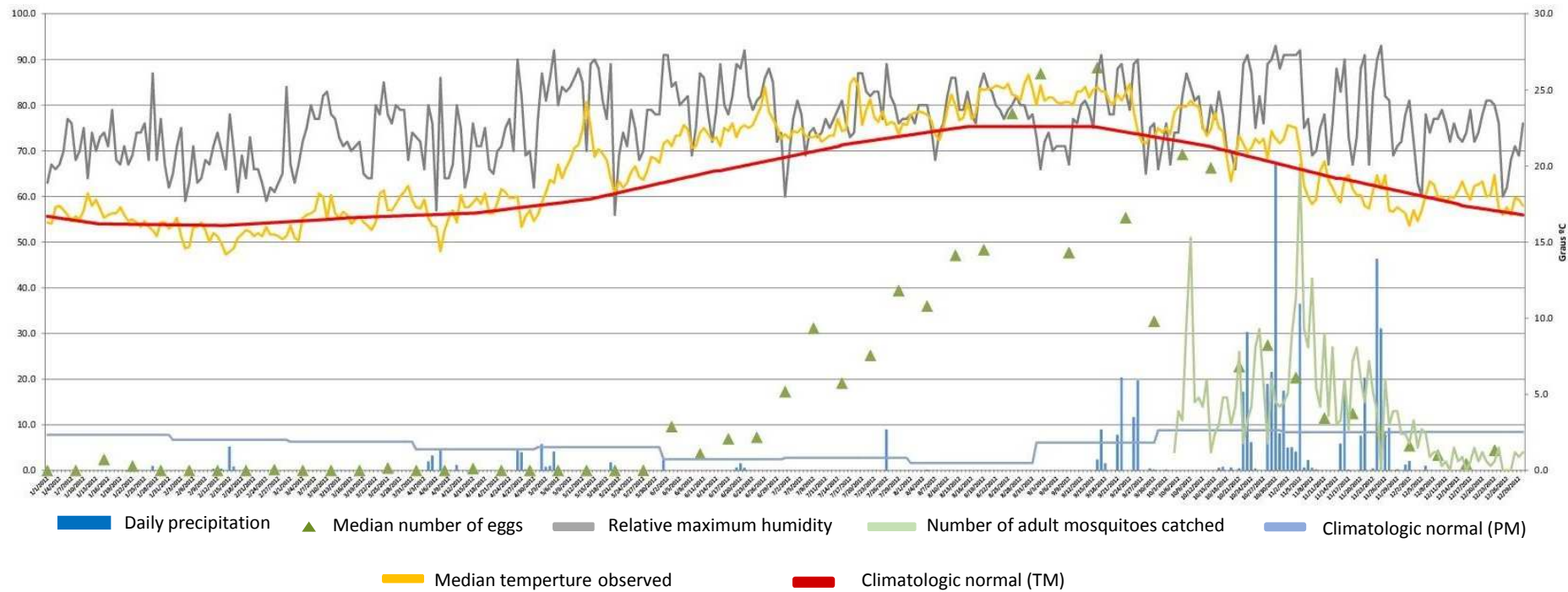

Supplement: Additional file 8 — Variation of the temperature, humidity and precipitation from September 2011 to July 2012 in Madeira Island. [file 1471-2458-14-39-S8.pdf]
