# Supplementary material for: Strengthening the perception-assessment tools for dengue prevention: a cross-sectional survey in a temperate region (Madeira, Portugal)
Source: BMC Public Health. 2014 Jan 15;14:39. doi: 10.1186/1471-2458-14-39 (PMC3905660; doi:10.1186/1471-2458-14-39)
Supplement: Additional file 2 — Relevance of cumulative knowledge. Exploring why a ‘higher’ level of knowledge doesn’t necessarily reflect a ‘better’ awareness. [file 1471-2458-14-39-S2.pdf]

Let’s explore the awareness of two hypothetic cases, respondent number 209 and number 344. Number 209 knew that mosquitoes could transmit disease and gave “Dengue” as an example, showing that he/she had a complete notion of the Medical Importance of Mosquitoes; he/she also knew that those mosquitoes are in Santa Luzia (his/her area of residence), and recognized the possibility of a dengue outbreak in Madeira. He/she had only understood two of the five themes assessed (this knowledge corresponds to a Score of EP = 4). Case number 344, had the maximum score for four of the five analysed themes, he/she admitted more concepts than number 209. He/She simply did not admit that mosquitoes could breed inside houses (this knowledge corresponds to a Score of EP=9). Number 344 probably won’t adhere to the domestic control since he/she didn’t understand the real need of control his/her domestic area. Even though case number 344 has more essential perceptions than case number 209, none of them have the sufficient amount of knowledge to be aware of their own involvement in domestic vector control.
