# Supplementary material for: Strengthening the perception-assessment tools for dengue prevention: a cross-sectional survey in a temperate region (Madeira, Portugal)
Source: BMC Public Health. 2014 Jan 15;14:39. doi: 10.1186/1471-2458-14-39 (PMC3905660; doi:10.1186/1471-2458-14-39)
Supplement: Additional file 3 — Myth’s appearance. Explaining an example of how a myth can appear from a partial (non-cumulative) understanding. [file 1471-2458-14-39-S3.pdf]

For instance, let’s explore the meaning of (correctly) admitting that water accumulation leads to the breeding of mosquitoes, but also (erroneously) believe that “food debris can contribute to mosquito breeding”. Food debris on its own (without water accumulation) does not serve as a larvae habitat. Without the mentioning of water accumulation, this belief supports the erroneous idea that “clean places aren’t infested by mosquitoes”. As a result, people who assume their own houses as being “clean” may not feel implicated in domestic *aegypti*-control.
