# Supplementary material for: Strengthening the perception-assessment tools for dengue prevention: a cross-sectional survey in a temperate region (Madeira, Portugal)
Source: BMC Public Health. 2014 Jan 15;14:39. doi: 10.1186/1471-2458-14-39 (PMC3905660; doi:10.1186/1471-2458-14-39)
Supplement: Additional file 4 — False perceptions/myths estimation through the analysis of residents’ topic understanding. [file 1471-2458-14-39-S4.pdf]

Concepts acknowledment comparison			Analysis of Topic Understanding	Community Understanding / Alleged myths
**Concept 1**	**Concept 2**		**Medical Importance**	
✓	✓	31·9 **%** (377 ind.)	Residents admitted that mosquitoes transmit diseases such as 'Dengue' (22·5%), 'Malaria' (9·5%), 'Yellow fever' (3·1%) or other mosquito-borne diseases (1·2%) or few of the latters.	Residents seemed to understand the real medical importance of mosquitoes and, thus the relevance of being involved in the *aegypti*-control.
✓	X	54·4 **%** (643 ind.)	Even though admitting that mosquitoes can transmit diseases, these residents did not know what kind of diseases do mosquitoes transmit. Some residents erroneously referred 'allergies' as mosquito-transmitted diseases (6.3%) and 4·5% mentioned other false clinical consequences such us 'SIDA', 'fever' or 'cancer'.	These residents were not aware of the relevance of being involved in the *aegypti*-control. Alledged Myth 1: "Mosquitoes only cause mild clinical consequences such as allergies, fever, etc".
X	✓	Not observed		
X	X	13·7**%** (162 ind.)	Residents did not know that mosquitoes can transmit diseases	Residents did not understand the medical importance of mosquitoes. Alleged Myth 2: "Mosquitoes do not transmit diseases"
**Concept 3**	**Concept 4**	**%**	**Local Risk**	
✓	✓	15·4**%** (182 ind.)	Residents recognized that there were mosquitoes that transmit diseases in their residential area, and, also, that there was a risk of a dengue outbreak in Madeira.	Residents seemed to understand the local risk they are submitted and, thus the urgency of being involved in the *aegypti*-control.
✓	X	18·8 **%** (222 ind.)	Residents recognized the presence of mosquitoes that transmit diseases in their residential area; however they believed that a dengue outbreak will not emerge in the island. Alledgedly some made this confusion because they did not recognize dengue as a mosquito-borne disease (20·3%). Eventually some residents could think that Madeira is "protected" since those kind of severe epidemic diseases historically never occured in temperate countries.	These residents were not aware of the the urgency of being involved in the *aegypti*-control. Alledged myths 3 and 4: (i) – "Dengue is not a mosquito-borne disease"; (ii) - "Dengue only occur in tropical/non-develloped countries".
X	✓	15·9 **%** (188 ind.)	Residents did not recognize the presence of mosquitoes, in their residential area, that can transmit diseases; but admitted that a dengue outbreak can emerge in the island. These residents did not have a correct notion of the *aegypti's* distribution area. Since 22·2% out of these group referred not be 'biten by mosquitoes', they could believe that they are at lower risk of being infected in an eventual outbreak.	Residents did not understand the risk they are subjected to and neither the urgency of being involved in the *aegypti*-control. Alledged myths 5 and 6: (i) -"Mosquitoes are allocated in a specific area and are not able to spread through the island"; (ii) - "Since I do not feel the byte, I am not a risk of being biten/infected".
X	X	49·9 **%** (590 ind.)	Residents did not recognize mosquitoes that transmit diseases in their residential area neither the possibility of a dengue outbreak in the island.	Residents did not understand the risk they are subjected to neither the urgency of being involved in the *aegypti*-control. Alleged Myth 7 : "Madeira's residents are not at risk"
**Concept 5**	**Concept 6**	**%**	**Domestic Attribute**	
✓	✓	20·0 **%** (236 ind.)	Residents know that mosquitoes can breed inside houses and recognized that domestic *aegypti*-control do have impact in the reduction of *aegypti*-population.	Residents seemed to understand the domestic attribute of the *aegypti*-control and, thus why community is the key intervenient in the *aegypti*-control.
✓	X	6·4**%** (76 ind.)	Residents know that mosquitoes can breed inside houses but they did not believe that the domestic *aegypti*-control have impact in the reduction of the *aegypti*'s population. They probably believed that other intervenients have much more impact in the reduction of the *aegypti*'s population.	Residents did not understand the domestic attribute of the *aegypti*-control, neither why community is the key intervenient in the *aegypti*-control. Alledged Myth 8: "Local health authorities are the key intervenient in the control of mosquitoes ".

X	✓	45·9 **%** (543 ind.)	Mosquitoes cannot breed inside houses but domestic *aegypti*-control do have impact in the reduction of *aegypti*-population in the neighborhood. Those respondents believed in their role in domestic *aegypti*-control but did not understood why that control has an impact.	Residents did not understand the the domestic attribute of the *aegypti*-control, neither why community is the key intervenient in the *aegypti*-control. Alledged Myth 9: "Other protective measures can control mosquitoes".
X	X	27·7**%** (327 ind.)	Residents do not know that mosquitoes transmit disease, neither that their involvement have an impact in the control of mosquitoes.	Residents did not understand the domestic attribute of the *aegypti*-control, neither why community is the key intervenient in the *aegypti*-control. Alleged Myth 10: " I am (Community is) not an intervenient in the *aegypti*-control".
**Concept 7**	**Concept 8**	**%**	**Mosquito Breeding**	
✓	✓	27·6 **%** (326 ind.)	Residents only identifyied water-containers (and not other false issues) as mosquitoes' breeding contributors.	Residents seemed to understand where do mosquito breed and, thus the need of the *aegypti*-control activities.
✓	X	46·5**%** (550 ind.)	Residents identifyied water-containers but also other false issues (food debris and pets) as mosquitoes' breeding contributors. These residents did not comprehend what lead to the breeding of new mosquitoes and, thus did not understand the proposed measures to control them.	Residents seemed to not understand where mosquitoes breed and neither the need of the *aegypti*-control activities. Alledged Myths 11 and 12: "Clean houses or houses without pets/animals do not have mosquitoes" or "Clean people did not need to be involved in mosquito control".
X	✓	12·0 (142 ind.)	Residents did not identify water-containers neither other false issues (food debris and pets) as mosquitoes' breeding contributors. These residents did not know where do mosquitoes breed.	Residents seemed to not understand where mosquitoes breed and neither the need of the *aegypti*-control activities.
X	X	13·9 (164 ind.)	Residents did not identify water-containers but did identify other false issues (food debris and pets) as mosquitoes' breeding contributors	Residents are completely mistaken regarding mosquitoes breeding and, thus did not uderstand the need of the *aegypti*-control activities. Alledged Myths 11 and 12: "Clean houses or houses without pets/animals do not have mosquitoes" or "Clean people did not need to be involved in mosquito control".
**Concept 9**	**Concept 10**	**%**	**Control Measures**	
✓	✓	13·0 (154 ind.)	Residents only recognized water-containers removal (and not other false measures) as "effective to control mosquitoes"	Residents seemed to recognize effective control measures and, thus understand how the domestic *aegypti*-control should be done.
✓	X	64·1 (758 ind.)	Residents recognized water-containers removal and also other false measures (such as insecticide indoor application and flyswatter use) as "effective to control mosquitoes"	Residents seemed to not be focused on effective control measures and, thus did not understand how the domestic *aegypti*-control should be done. Alledged Myth 13: "Using insecticides or the flyswatter, I am already contributing to control the *aegypti*-mosquito"
X	✓	7·3 (86 ind.)	Residents did not recognize water-containers removal neither other false measures (such as insecticide indoor application and flyswatter use) as "effective to control mosquitoes". These residents did not know how to control mosquitoes.	Residents not recognized effective control measures and did not understand how domestic *aegypti*-control shou done.

X	X	15·6 (184 ind.)	Residents recognized water-containers removal and also other false measures (such as insecticide indoor application and flyswatter use) as “effective to control mosquitoes”	Residents seemed to not be focused on effective control measures and thus did not understand how the domestic aegypti-control should be done.<br>Alleged Myth 13: “By using protective meausres (such as insecticides or the flyswatter), I am already contributing to control the *aegypti*-mosquito”

n TOTAL (Scored Population) = 1182 individuals
