# Supplementary material for: Strengthening the perception-assessment tools for dengue prevention: a cross-sectional survey in a temperate region (Madeira, Portugal)
Source: BMC Public Health. 2014 Jan 15;14:39. doi: 10.1186/1471-2458-14-39 (PMC3905660; doi:10.1186/1471-2458-14-39)
Supplement: Additional file 6 — Domestic breeding sites predictors. Associations/differences with socio-demographic data. [file 1471-2458-14-39-S6.pdf]

	Residents living in houses WITH BREEDING SITE(S) n total = 1018		Residents living in houses WITHOUT BREEDING SITE(S) n total = 261		
	**n**	**%**	**n**	**%**	***p*-value**
**Gender**					0·665
Male	406	40·2	100	38·8	
Female	603	59·8	158	61·2	
**Education level (years)**					0·007
Never studied (0)	62	6·2	13	5·1	
Fourth Grade (4)	402	40·4	82	32·2	
Ninth Grade (9)	226	22·7	55	21·6	
High School (12)	171	17·2	49	19·2	
Upper Education (+12)	135	13·6	56	22·0	
**Age groups (years old)**					0·002
25 or younger	147	14·7	23	9·0	
26-35	144	14·4	28	11·0	
36-45	145	14·5	52	20·4	
46-55	176	17·6	45	17·6	
56-65	156	15·6	26	10·2	
66-75	137	13·7	48	18·8	
76 or older	96	9·6	33	12·9	
**Municipal Division**					<0·001
Santa Luzia	281	27·6	136	52·3	
São Pedro	271	26·6	43	16·5	
Câmara de Lobos	466	45·8	81	31·2	
**Travelled to EC***					0·204
Yes	240	24·2	71	28·1	
no	752	75·8	182	71·9	
**Biten by mosquitoes**					0·273
yes	744	73·6	200	76·9	
No	26	26·4	60	23·1	

*Dengue Endemic Countries ; ‘ chi-square test (Pearson test)
