# Supplementary material for: Strengthening the perception-assessment tools for dengue prevention: a cross-sectional survey in a temperate region (Madeira, Portugal)
Source: BMC Public Health. 2014 Jan 15;14:39. doi: 10.1186/1471-2458-14-39 (PMC3905660; doi:10.1186/1471-2458-14-39)
Supplement: Additional file 7 — Multiple regression model predicting socio-demographic determinants to achieve at least seven perceived essential concepts (EP-score equal to or higher than seven). [file 1471-2458-14-39-S7.pdf]

	**OR and 95% CI:**	Lower	Upper	***p*-value**
**Constant**	1·456	-	-	0·385
**Gender**				
Male	0·579	0·419	0·800	0·001
Female a	-	-	-	-
**Education level (years)**				
Never studied (0)	28·940	6·490	129·047	<0·001
Fourth Grade (4)	11·425	6·662	19·590	<0·001
Ninth Grade (9)	4·370	2·717	7·030	<0·001
High School (12)	2·116	1·357	3·302	0·001
Upper Education (+12) a	-	-	-	-
**Age groups (years old)**				
25 or younger	1·676	0·753	3·733	0·206
26-35	0·874	0·419	1·820	0·718
36-45	0·700	0·349	0·402	0·314
46-55	0·712	0·759	1·411	0·330
56-65	0·730	0·359	1·481	0·383
66-75	0·919	0·453	1·865	0·816
76 or older a	-	-	-	-
**Municipal Division**				
Santa Luzia	0·479	0·304	0·753	0·001
São Pedro	0·701	0·445	1·104	0·126
Câmara de Lobos a	-	-	-	-
**Travelled to EC****				
Yes a	-	-	-	-
No	1·174	0·817	1·688	0·385
**Biten by mosquitoes**				
Yes a	-	-	-	-
No	1·789	1·189	2·693	0·005

*Dengue Endemic Countries; a Reference
