# Supplementary material for: Strengthening the perception-assessment tools for dengue prevention: a cross-sectional survey in a temperate region (Madeira, Portugal)
Source: BMC Public Health. 2014 Jan 15;14:39. doi: 10.1186/1471-2458-14-39 (PMC3905660; doi:10.1186/1471-2458-14-39)
Supplement: Additional file 9 — Representation of the aegypti-infestation pattern found in the domestic regions of AEGYPTI-area in Madeira Island. [file 1471-2458-14-39-S9.pdf]

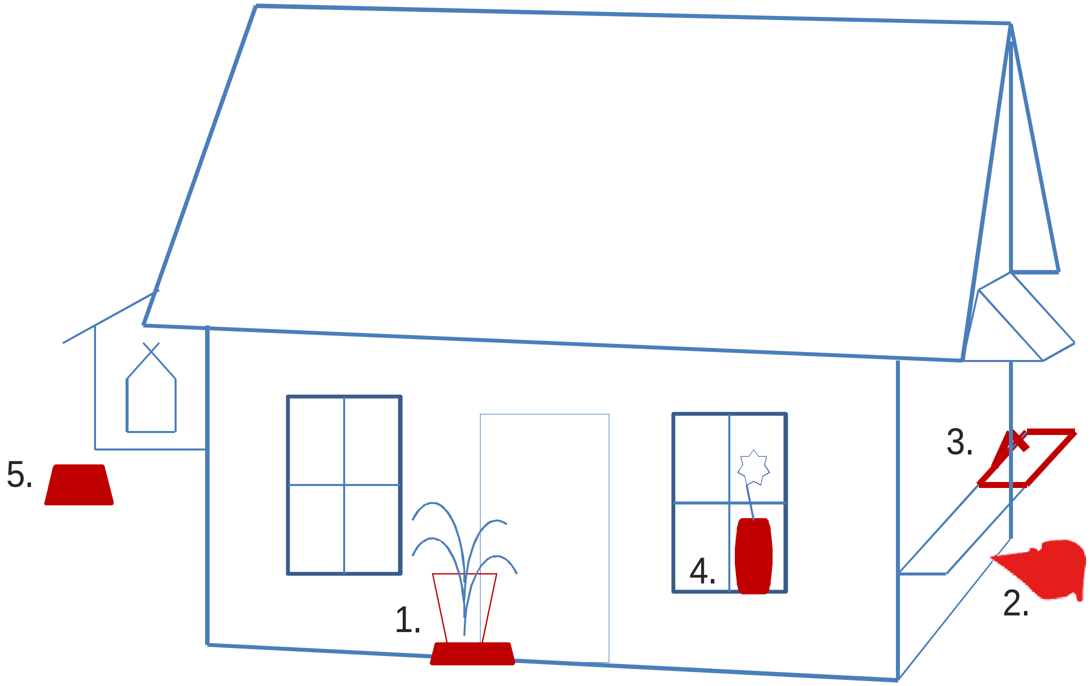

1. Flower-pot dish (52·7%)
2. Water-accumulation on deck* (35·7%)
3. Outdoor sink **(23·3%)
4. Flower vases (21·7%)
5. Pets water dish (18·8%)

* Floor slope;
uneven floor;

** outdoor kitchen;
clothe water tank; floor drains
